# Expanding the Spectrum: a case of Giant cell arteritis encountered in familial Mediterranean fever

**DOI:** 10.1093/omcr/omae197

**Published:** 2025-02-22

**Authors:** Rita D Moncayo, Mukhammad B Sultanov, May T H Tun, Fatemeh Mohammadrezaei, Omair A Khan, Diana Gutierrez, Sarita Konka

**Affiliations:** Department of Internal Medicine, Maimonides Medical Center, 4802 Tenth Avenue, Brooklyn, NY 11219, Unites States of America; Department of Internal Medicine, Maimonides Medical Center, 4802 Tenth Avenue, Brooklyn, NY 11219, Unites States of America; Department of Internal Medicine, Maimonides Medical Center, 4802 Tenth Avenue, Brooklyn, NY 11219, Unites States of America; Department of Internal Medicine, Maimonides Medical Center, 4802 Tenth Avenue, Brooklyn, NY 11219, Unites States of America; Department of Internal Medicine, Maimonides Medical Center, 4802 Tenth Avenue, Brooklyn, NY 11219, Unites States of America; Department of Internal Medicine, Maimonides Medical Center, 4802 Tenth Avenue, Brooklyn, NY 11219, Unites States of America; Department of Rheumatology, Maimonides Medical Center, 4802 Tenth Avenue, Brooklyn, NY 11219, United states of America

**Keywords:** Familial Mediterranean Fever, Monogenic fever, vasculitis, MEFV Gene, Inflammation

## Abstract

Familial Mediterranean Fever (FMF) is an autoinflammatory genetic disorder causing recurrent fever episodes due to MEFV gene mutations, typically affecting small and medium-sized vessels. We present a 69-year-old male with FMF exhibiting features suggestive of large vessel vasculitis, challenging the conventional understanding of FMF. The patient had an extended fever episode, a self-resolving groin rash, intermittent diarrhea, and a decline in health over six months. A PET scan showed increased uptake in large vessels, especially in the bilateral lower extremities, indicating possible large vessel vasculitis. The patient's positive response to corticosteroid therapy supported an inflammatory etiology. This case underscores the need for a broader differential diagnosis in atypical FMF presentations and suggests further research into the relationship between FMF and large vessel vasculitis.

Familial Mediterranean fever (FMF) is an autoinflammatory genetic disorder causing recurrent fevers and serosal inflammation of the abdomen, lungs, and joints [1]. Several studies have reported an increased incidence of vasculitis in FMF patients, namely IgA vasculitis, polyarteritis nodosa and Behçet’s disease [2].

In this study, we present a unique case challenging FMF’s conventional understanding. This case demonstrates an overlap with clinical features suggestive of large vessel vasculitis (LVV).

A 69-year-old male with a past medical history of Beta Thalassemia minor, Familial Mediterranean Fever on colchicine, hypertension, hypothyroidism, and chronic kidney disease presented to the emergency department with 10 days of fever.

Accompanying symptoms included fatigue, diaphoresis, and anorexia. He denied any recent exposures, travel, or insect bites. Interestingly, he reported a self-resolving groin rash 5–6 days prior to presentation. He experienced intermittent diarrhea without any alarming features. He emphasized that these episodes were distinct from his typical FMF flares, which he hadn’t experienced in recent years. Over the past six months, he reported a gradual decline in his overall health, manifesting as fatigue, decreased appetite, lethargy, and an unintentional weight loss of approximately 10 lbs. Physical examination revealed no signs of acute joint inflammation, with peripheral pulses intact in all extremities.

Initial laboratory investigations revealed an elevated ESR at 73 mm/hr, CRP of 17.248 mg/l, and ferritin of 321.6 ng/ml, suggestive of a systemic inflammatory response. Other notable findings included anemia with a hemoglobin of 9.1 g/dl and microcytosis (MCV 62.3 fL), thrombocytopenia with a platelet count of 129 x 10^9/L, and renal impairment with a creatinine of 2.2 mg/dl. Urinalysis was positive for proteinuria. Septic work up came back negative, including the Karius test. Serologic tests were negative for tuberculosis, hepatitis, CMV, EBV, and HIV.

Imaging studies, including a non-contrast chest CT, revealed small bilateral pleural effusions, pulmonary nodules, atherosclerotic changes in the thoracic aorta, and splenomegaly. An echocardiogram was essentially normal with an ejection fraction of 61%. Further rheumatological workup, including ANA, ANCA, rheumatoid factor, complement levels, and protein/urine electrophoresis, was unremarkable. A bone marrow biopsy did not show any abnormalities.

Given the clinical suspicion of vasculitis, a positron emission tomography (PET) was performed, which demonstrated increased uptake in the bilateral femoral arteries, raising the possibility of large vessel vasculitis, particularly Giant cell arteritis (GCA) (Fig. 1). Although the patient did not exhibit classical symptoms of temporal arteritis, bilateral temporal artery biopsies were performed and were negative for GCA.

**Figure 1:**
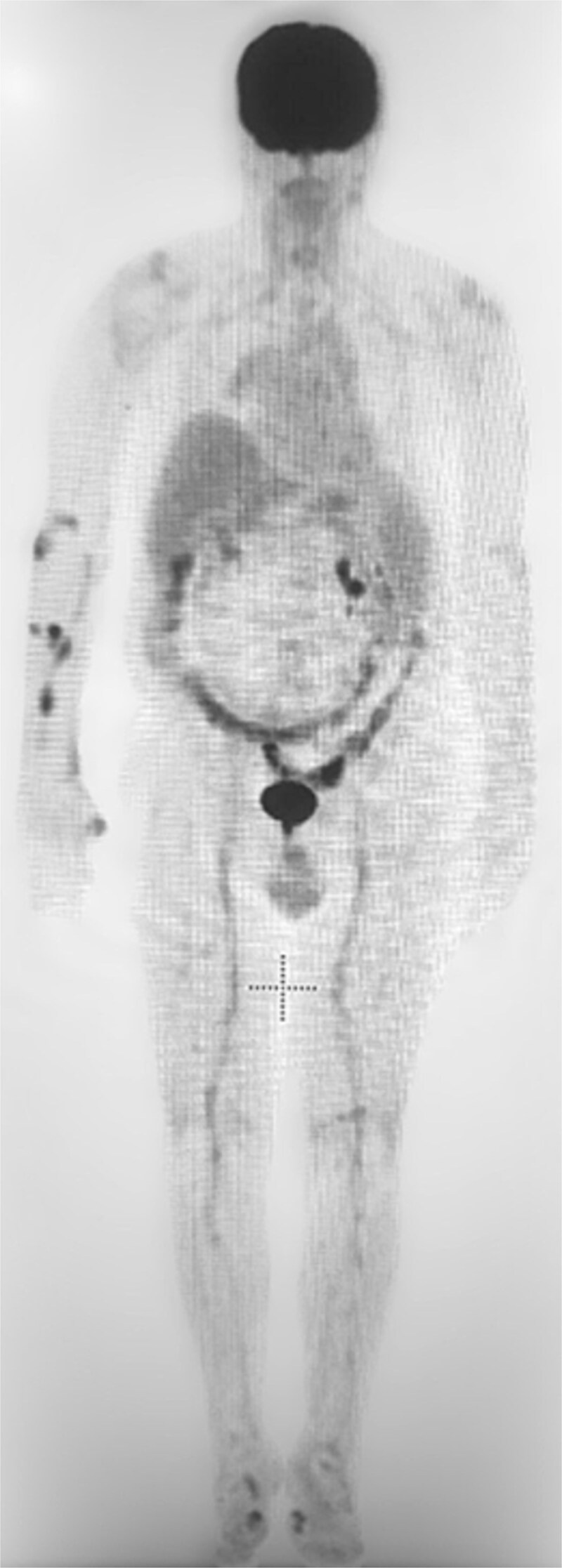
There is increased uptake throughout the large vessels in the bilateral lower extremities extending from the femoral vessels distally into the legs.

The patient was initiated on prednisone 60 mg daily, with a marked improvement in his symptoms. He was discharged on the same dose with a plan for outpatient follow-up. During his first outpatient visit, a renal biopsy was recommended to rule out small vessel vasculitis and amyloidosis, both known associations with FMF. The biopsy revealed global glomerulosclerosis and changes suggestive of chronic hypertensive and age-related renal injury.

During follow-up visits, attempts to reduce prednisone to 20 mg daily led to a recurrence of fever. Although adding tocilizumab was considered to manage the inflammation, the patient chose not to proceed with it. Instead, the patient responded well to a more gradual tapering of prednisone. Currently, patient is stable on a low maintenance dose of 2.5 mg of prednisone daily, along with colchicine.

FMF is the most prevalent monogenic periodic fever syndrome. The association with LVV is a rare occurrence. LVV comprises two distinct conditions, GCA and Takayasu arteritis (TAK) [2].

Two case reports by Zihni et al. [3] and Alibaz et al. [4], reported a rare co-existence of FMF and TAK. These reports outlined cases with male patient presentations demonstrating pulselessness while on colchicine therapy for FMF. Radiographic assessments revealed occlusion in either the carotid arteries or subclavian arteries. The primary course of treatment for these cases involved methylprednisolone and azathioprine. Having refractory clinical courses, with the latter case responding to Infliximab.

Malik et al. [5], describe a case of FMF associated with aortic dissection in a young male. With evidence of a chronic inflammatory process on aortic histological examination.

Zenone et al. [6], reported a case of FMF associated with Cogan syndrome in a middle-aged male complicated with Aortitis that responded to prednisone.

The prevalence of upper and lower extremity vasculitis related to GCA was estimated to be 26% and 18%, respectively. Large vessel involvement was evidenced in 27% to 67% by angiography, 83% by PET imaging and 100% by autopsy. Comparing the involvement of iliofemoral arteries in LVV subtypes, GCA has 13%–47% and TAK has 16%–20% [7]. Based on this evidence, we can suspect that the vasculitic features of our patient are similar to those seen in GCA.

This case report delineates a rare co-occurrence of FMF with LVV, notably the first to associate FMF with GCA, expanding our understanding of FMF beyond its conventional link to smaller vessel vasculitis.
