# Correction to: Expanding the Spectrum: a case of Giant cell arteritis encountered in familial Mediterranean fever

**DOI:** 10.1093/omcr/omaf098

**Published:** 2025-06-27

This is a correction to: Rita D Moncayo, Mukhammad B Sultanov, May T H Tun, Fatemeh Mohammadrezaei, Omair A Khan, Diana Gutierrez, Sarita Konka, Expanding the Spectrum: a case of Giant cell arteritis encountered in familial Mediterranean fever, *Oxford Medical Case Reports*, Volume 2025, Issue 2, February 2025, omae197, https://doi.org/10.1093/omcr/omae197

The original version of the manuscript was erroneously published without the **Abstract** section. This error is emended and the **Abstract** section added to the paper. In **Keywords**, “Inflammationinflammation” is emended to read “Inflammation”.

